# Impact of meteorological and environmental factors on the spatial distribution of *Fasciola hepatica* in beef cattle herds in Sweden

**DOI:** 10.1186/s12917-015-0447-0

**Published:** 2015-06-09

**Authors:** Adam Novobilský, Jakub Novák, Camilla Björkman, Johan Höglund

**Affiliations:** Department of Biomedical Sciences and Veterinary Public Health, Section for Parasitology, Swedish University of Agricultural Sciences (SLU), Box 7036, 75007 Uppsala, Sweden; Department of Geography, Tartu University, Vanemuise 46, 51 014 Tartu, Estonia; Department of Social Geography and Regional Development, Charles University in Prague, Faculty of Science, Albertov 6, 128 43 Prague, Czech Republic; Department of Clinical Sciences, Swedish University of Agricultural Sciences (SLU), Box 7054, 75007 Uppsala, Sweden

**Keywords:** Antibodies, ELISA, Environmental, Epidemiology, GIS, Galba truncatula, Rainfall, Scandinavia, Risk analysis, Regression

## Abstract

**Background:**

*Fasciola hepatica* is a parasite with a significant impact on ruminant livestock production. Previous studies in north-west Europe have described its geographical distribution and determined potential predictors of fasciolosis using geographical information system (GIS) and regression modelling. In Sweden, however, information about the distribution of fasciolosis is limited. This study examined the geographical distribution of *F. hepatica* and identified high-risk areas for beef cattle in Sweden and sought to characterise potential predictors. Beef cattle serum samples were collected during winter 2006-2007 from 2135 herds which were examined for *F. hepatica* antibodies by enzyme-linked immunosorbent assay (ELISA). Fasciolosis distribution maps were created using GIS based on postcode location of seropositive herds. Spatial scan analysis (SaTScan) was performed to determine high-risk areas. Using datasets on animal density, temperature, precipitation and Corine land cover data, including soil type and soil mineral concentrations in Sweden, bivariate and multiple logistic regression analyses were carried out in R software to reveal potential predictors of *F. hepatica* infection.

**Results:**

Overall herd seroprevalence of *F. hepatica* in beef cattle was 9.8 % (95 % CI: 8.6-11.1). An irregular spatial distribution of *F. hepatica,* with two main clusters, was observed in south-west Sweden. The most northerly occurrence of *F. hepatica* in the world was documented. The final model explained 15.8 % of the variation in *F. hepatica* distribution in study herds. Absence of coniferous forest was the variable with the highest predictive value. Precipitation in July-September, Dystric Cambisol, Dystric Regosol, and P and Cu concentrations in soil were other negative predictors. Beef cattle herd density, Dystric Leptosol and Fe concentration were positive predictors.

**Conclusions:**

The spatial distribution of *F. hepatica* in Swedish beef cattle herds is influenced by multi-factorial effects. Interestingly, absence of coniferous forest, herd density, specific soil type and concentration of some soil minerals are more important predictors than climate factors.

**Electronic supplementary material:**

The online version of this article (doi:10.1186/s12917-015-0447-0) contains supplementary material, which is available to authorized users.

## Background

Fasciolosis is a parasitic infection of ruminants worldwide caused by the common liver fluke *Fasciola hepatica* [1]. The main impact in cattle production is due to reduced weight gain and poor carcass status and condemnation of livers at slaughter [1, 2]. In the past decade, the prevalence of bovine fasciolosis in some European countries has increased due to milder winters, improved sensitivity of diagnostic methods and/or failure of control [3].

Based on meat inspection data, the prevalence of fasciolosis in Swedish cattle was 3 % in 2005 but rose to almost 11 % in 2013 [4]. Analysis of the herd seroprevalence and spatial distribution in Sweden to date is limited to observations from abattoirs. Although abattoir data provide an estimate of the prevalence of *F. hepatica* in different geographical regions, it has been shown that approximately one-third of infected livers go undetected at meat inspection [5]. Detection of circulating specific antibodies against liver flukes by enzyme-linked immunosorbent assay (ELISA), in serum or milk samples, is currently an efficient method of monitoring fasciolosis [3, 6, 7]. Owing to the ease of collection of bulk-tank milk (BTM) samples, most studies to date have been conducted in dairy herds [8–13].

Under Swedish animal welfare legislation, all cattle older than six months, except bulls, must be allowed out to pasture in summer [14]. According to recent reports, problems with *F. hepatica* are common today in both conventional and organic dairy and beef units, particularly in cattle kept on wet lowland pastures in years when the weather conditions are favourable for parasite transmission. Beef cattle management in Sweden is characterised by long-term grazing and some farmers in southern Sweden have even introduced year-round grazing on pasture (U. Eliasson, personal communication 2014). Unlike dairy cows, beef cattle often graze marginal natural pasture with suitable habitats for the main intermediate host, the snail *Galba truncatula* [15].

Monitoring the spatial distribution of fasciolosis using Geographical Information System (GIS) allows identification of high-risk areas, enabling local effective control measures [8]. Furthermore, forecasting model maps can be generated by including environmental and climate data [16, 17]. Such spatial risk analyses in dairy herds have been performed in Belgium [18], Germany [11] and England, Wales [9] and recently also in Ireland [19, 20]. Proportion of grassed area and proportion of water bodies are reported to be the strongest predictors of *F. hepatica* infection in Germany [11]. In contrast, rainfall and temperature are reported to be the most important predictors in England, Wales and Ireland, along with soil structure and minerals [10, 19]. These factors are all associated with the habitat preferences of *G. truncatula* [17, 21, 22].

The aims of the present study were to conduct a nation-wide serological survey of *F. hepatica* in Swedish beef cattle and to perform regression analysis on some environmental and climate variables, in order to identify *F. hepatica* high-risk areas and characterise potential risk factors for *F. hepatica* exposure in beef cattle herds in Sweden.

## Methods

### Study design and sampling

Blood samples were collected from young beef cattle over 12 months of age within the Swedish Bovine Viral Diarrhoea (BVDV) surveillance programme and have been used previously to investigate the distribution of *Neospora caninum* in Sweden [23]. Thus, the sampling process was created primarily for BVDV surveillance and approved by the Swedish Board of Agriculture in accordance with the national legislation in Sweden (Animal Welfare Act 2009/021). Every 12^th^ sample was systematically selected from samples submitted between November 2006 and May 2007, yielding a total of 2767 serum samples from 2135 herds. All samples were accompanied by herd identification by postcode. The herds sampled represented approximately 20 % of all Swedish beef herds at the time of sampling and 1-5 samples were collected per herd [23]. All sampled animals were grazed for one season, in 2006.

### Serology

Serum antibody levels for *F. hepatica* were determined using an in-house excretory/secretory (ES), antigen-specific, enzyme-linked immunosorbent assay (ELISA). The *F. hepatica* ES antigen preparation and ELISA protocol were carried out as described previously [24]. The ELISA results were expressed as percentage positivity (PP), where PP = (mean OD of tested sample (*n* = 2)/mean OD of the positive control) * 100. As a positive control, serum samples originating from naturally infected beef cows were used [25]. Samples with PP ≥15 % were considered positive, a level determined previously by receiver operating characteristic (ROC) curve analysis [25], where the sensitivity was 96 % (95 % CI: 88-100) and the specificity 98 % (95 % CI: 94-100).

### Data analysis

#### Spatial data

Swedish five-digit postcode data from 2007 were obtained from Statistics Sweden (Statistiska Centralbyrån; SCB). A postcode area (PSA) layer was constructed based on area polygons. In total, 1887 herds (88.4 % of herds included) were located based on their five-digit postcode area (5-PSA). Precise location information was lacking for 248 herds (11.6 %) and these were located by three-digit postcodes (3-PSA). A herd containing at least one positive serum sample was designated as seropositive. Seroprevalence was calculated as the number of positive herds out of the total number of herds included. Seroprevalence was determined for 3-PSA, municipality and county. Spatial clustering of positive and negative herds was then tested with the spatial scan statistic by the Bernoulli model [26], using SaTScan software version 9.2 (www.satscan.org). Using the Bernoulli model, clusters with increased relative risk were identified.

#### Variables tested

Data on beef cattle distribution, including number of herds and number and density of cattle at municipal and county level in 2007, were obtained from the Swedish Board of Agriculture [27]. Climate data were obtained from the Swedish University of Agricultural Sciences (SLU) GIS database, which was originally established and previously updated by the Swedish Meteorological and Hydrological Institute (SMHI). A dataset containing mean monthly temperature and precipitation for the period 1999-2009 was created. Average values were then calculated quarterly for temperature (Tem Q1, Q2, Q3, Q4) and precipitation (Pre Q1, Q2, Q3, Q4). Data on landscape properties and their spatial proportions in Sweden (in 2006) were taken from the Corine Land Cover dataset of the European Environment Agency (http://www.eea.europa.eu/data-and-maps) and included pasture (SC 231), complex cultivation patterns (SC 242), land principally occupied by agriculture with significant areas of natural vegetation (SC 243), broad-leaved forest (SC 311), coniferous forest (SC 312), mixed forest (SC 313), natural grasslands (SC 321), moors and heathland (SC 322), inland marshes (SC 411), peat bogs (SC 412), water courses (SC 511) and water bodies (SC 512). Data on soil and geological characteristics were obtained from the Geological Survey of Sweden (SGU) and the European Soil Portal (http://eusoils.jrc.ec.europa.eu/ESDB_Archive/ESDB/index.htm). The following variables were selected for analysis: soil type, pH and soil concentrations of six minerals (Ca, Cl, Cu, Fe, Mg and P). Vector and raster layers for all variables were created by ArcGIS version 9.2 (ESRI, USA). Data for regression analyses were extracted from these layers using ArcTools at 3-PSA level.

All regression analyses were carried out in R software (http://www.r-project.org/). In the initial bivariate analysis, all above-mentioned variables were tested against ELISA results at herd level (Additional file 1: Table S1). Significant variables (*P* ≤ 0.05) from the bivariate test were then further examined in multivariable models. Four different groups of variables were selected for multivariate modelling: temperature (Tem Q1, Q2, Q3, Q4 and cumulative temperature for the whole season); precipitation (Pre Q1, Q2, Q3, Q4 and cumulative precipitation for the whole season); all land cover variables (SC 231, 242, 243, 311, 312, 313, 321, 322, 411, 412, 511, 512); and soil pH and mineral content (Ca, Cl, Cu, Fe, Mg, P). Since beef cattle density did not fit in any of the models, this variable was included directly in the final model. For the final model, significant variables (*P* < 0.01) from the multivariate models and beef cattle herd density were selected.

## Results

### Spatial distribution

**Fig. 1:**
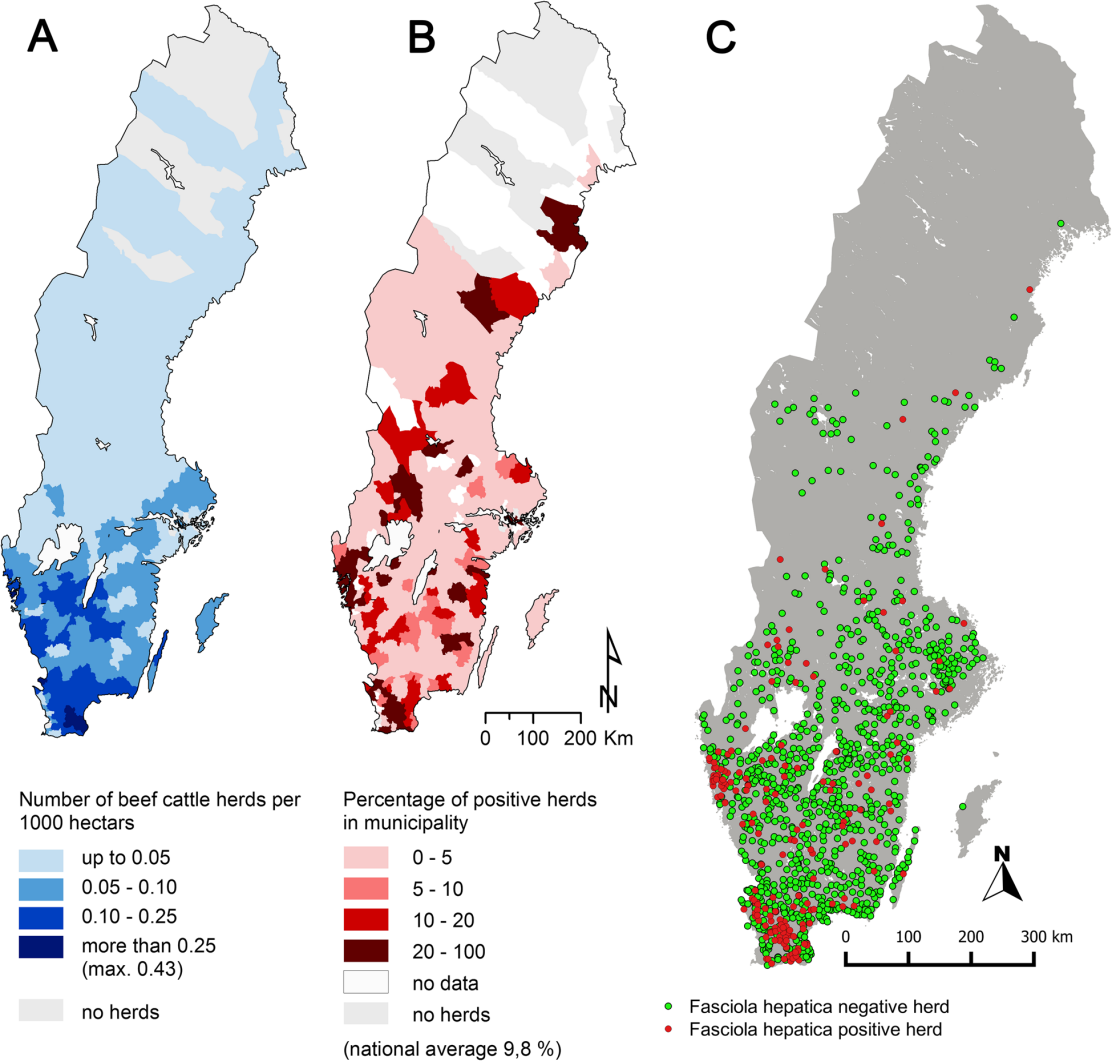
(**a**) Distribution of beef cattle herds, (**b**) herd seroprevalence of *Fasciola hepatica* at municipality level and (**c**) distribution of all herds examined in this study in Sweden

The overall seroprevalence of animals testing positive for *F. hepatica* antibodies was 9.5 % (*n* = 263/2767) in the 210 out of 2135 herds (9.8 %; 95 % CI 8.6 to 11.1 %) with seropositive animals. At the county level, the highest herd seroprevalence was found in Skåne (20.6 %), at the southern tip of Sweden, whereas no *F. hepatica*-positive herds were found on the island of Gotland in the Baltic Sea, in Örebro county in central Sweden or in Jämtland and Norrbotten in northern Sweden (Table 1). The most northerly *F. hepatica*-positive herd was found in Byske, on the Baltic shore of Västerbotten county (64°57.23070', 021°12.24984'). The spatial distribution of beef cattle herds in Sweden and of *F. hepatica*-positive herds is shown in Fig. 1.

**Table 1:** Basic characteristics of beef cattle distribution and seroprevalence in Sweden at county level

County	Number of herds	Number of beef cattle	Cattle density (per 1000 ha)	Number of examined herds per county	*Fasciola hepatica* prevalence (in %)
Stockholm	254	3489	2.09	60	3.3
Uppsala	421	5700	4.74	98	3.1
Södermanland	327	6163	7.03	64	3.1
Östergötland	669	13561	9.27	128	7.8
Jönköping	1098	13795	11.73	201	8.0
Kronoberg	709	8787	9.32	126	2.4
Kalmar	782	12347	5.99	130	3.9
Gotland	243	5409	3.53	28	0.0
Blekinge	440	5411	7.77	119	4.2
Skåne	1921	36187	21.14	408	20.6
Halland	675	8328	9.49	78	5.1
Västra Götaland	2231	29068	8.41	350	15.7
Värmland	533	8309	3.79	87	11.5
Örebro	364	5152	5.32	46	0.0
Västmanland	188	2701	4.75	32	3.1
Dalarna	371	4814	1.58	61	6.6
Gävleborg	393	5488	2.19	45	2.5
Västernorrland	335	4586	1.63	30	6.7
Jämtland	272	3800	0.70	37	0.0
Västerbotten	171	1619	0.24	6	16.7
Norrbotten	71	756	0.07	1	0.0
Sweden	12468	185470	3.49	2135	9.8

**Fig. 2:**
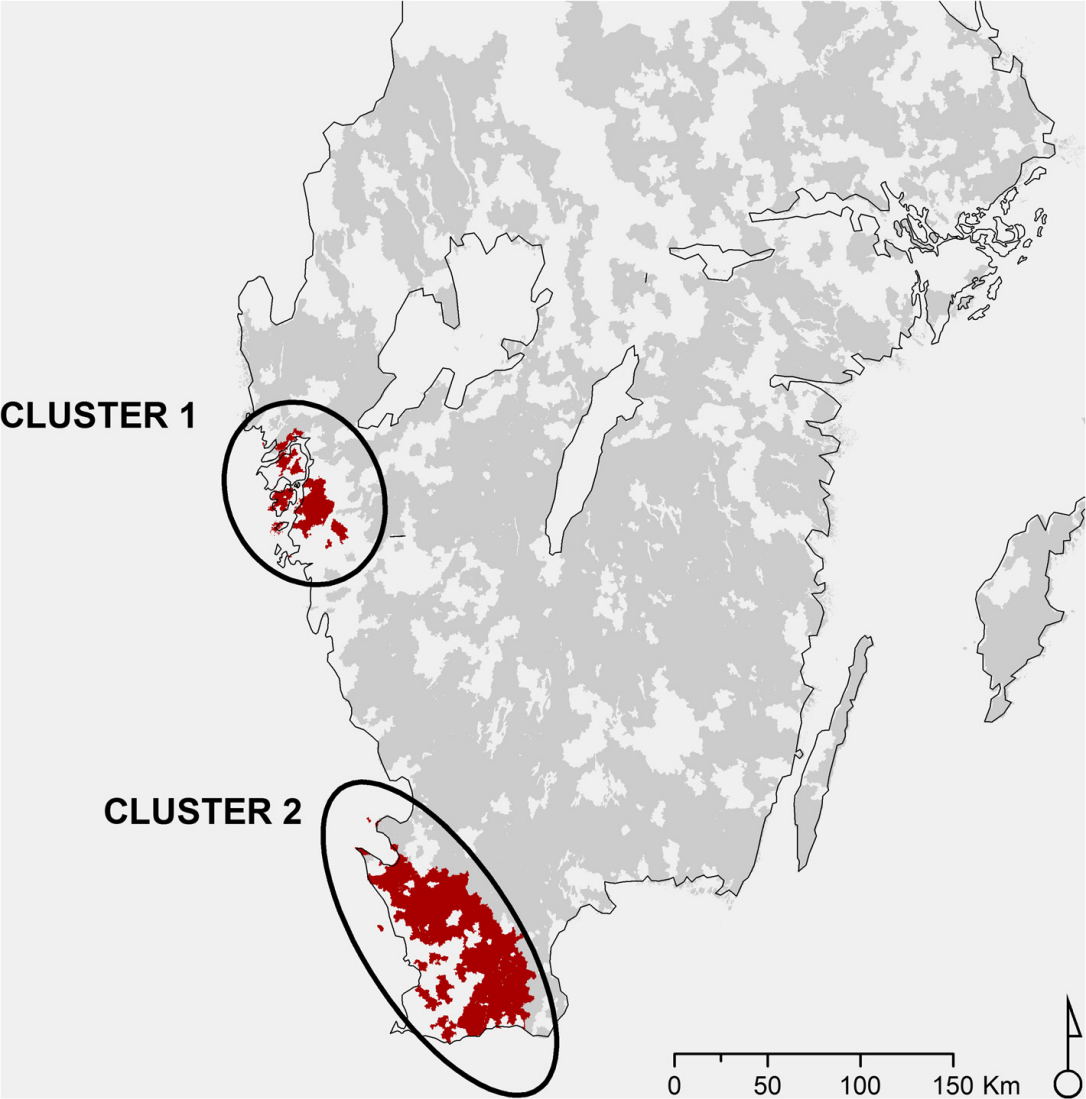
Spatial clusters in south-west Sweden with high seroprevalence of *Fasciola hepatica* as determined by spatial scan statistic (SaTScan software)

The spatial scan statistic revealed two significant (*P* = 0.001) clusters with a high risk of *F. hepatica*. Cluster 1, located on the western coast of Västra Götaland north of Gothenburg, contained 49 herds with a herd seroprevalence of 65 % (RR = 7.7; *P* = 0.001). Cluster 2, located in southern Skåne outside Malmö, contained 199 herds with a herd seroprevalence of 35 % (RR = 4.9; *P* = 0.001) (Fig. 2).

### Regression models

A total of 2030 PCA were arranged for regression models using ArcGIS version 10.2.2. For soil type and chemical composition, data were lacking for 433 PCA. Bivariate analyses of variables with *F. hepatica* seroprevalence data revealed positive correlations with rainfall and temperature, cattle and herd density, proportion of pasture per PCA, broad-leaved forest, Eutric Cambisol soil, Dystric Leptosol soil, and Fe and Mg concentrations in soil. Negative correlations were obtained for coniferous forest, water bodies and P and Cu soil concentrations (Additional file 1: Table S1).

The strongest correlation in the bivariate analyses was obtained for beef cattle herd density. In *Model Temperature*, average temperature in the second quarter of the year (Tem_Q2) was a significant positive predictor, whereas cumulative temperature during the entire year (Tem_season_sum) was negative (Additional file 2: Table S2). In *Model Precipitation*, snow/rainfall during the first quarter (Pre_Q1) was a positive predictor, whereas the third and fourth quarters (Pre_Q3; Pre_4) were negatively associated with herd seroprevalence (Additional file 3: Table S3). Degree of coverage with coniferous forest was the only significant negative predictor in *Model Corine Land Cover* (Additional file 4: Table S4). Of the six soil minerals investigated in *Model Minerals*, Cu and P concentrations were both negative predictors, while Fe was a positive predictor (Additional file 5: Table S5). The final multivariate model, obtained by manually controlled stepwise forward and backward selection of variables, explained 15.8 % of variation in the ELISA results (Table 2). The strongest negative predictor was coverage with coniferous forest. Positive predictors were beef cattle herd density, Dystric Leptosol and Fe concentration. Dystric Cambisol, Dystric Regosol, P and Cu concentrations were all negative predictors.

**Table 2:** Final model resulting from stepwise forward and backward manually controlled selection of variables

Variable	Estimate	S.E.	z value	Pr(>|z|)	Null deviance	Residual deviance	AIC	Pseudo- R^2^
Beef cattle herd density	3.497587	1.154833	3.029	0.002456 **	1073.59	942.64	968.64	0.158
SC 312 Coniferous forest	-0.021672	0.004828	-4.489	7.16e-06 ***				
tem_Q2	0.654361	0.616575	1.061	0.28856				
pre_Q1	0.01539	0.008364	1.84	0.06578				
pre_Q2	0.010399	0.006033	1.724	0.08475				
pre_Q3	-0.013316	0.006199	-2.148	0.03170 *				
pre_Q4	-0.008192	0.007002	-1.17	0.24199				
Soil type (ref. PZha, Haplic Podzol)								
ARha (Haplic Arenosol)	0.600641	0.39866	1.507	0.131901				
CMca (Calcaric Cambisol)	-0.472288	0.601682	-0.785	0.432485				
CMdy (Dystric Cambisol)	-0.932676	0.321899	-2.897	0.003763 **				
CMeu (Eutric Cambisol)	-0.626826	0.34534	-1.815	0.069509				
LPdy (Dystric Leptosol)	1.180656	0.340213	3.47	0.000520 ***				
RGca (Calcaric Regosol)	-0.086256	0.284393	-0.303	0.761662				
RGdy (Dystric Regosol)	-0.020017	0.006052	-3.307	0.000942 ***				
Fe (Iron)	0.580659	0.163602	3.549	0.000386 ***				
P (Phosphorus)	-5.443749	1.601878	-3.398	0.000678 ***				
Cu (Copper)	-0.051398	0.018112	-2.838	0.004542 **				

* Significant at 0.05 level; ** Significant at 0.01 level; *** Significant at 0.001 level

AIC: Akaike’s Information Criterion

## Discussion

To our knowledge, this is the first systematic serological survey of *F. hepatica* in beef cattle in Europe. The overall seroprevalence in Swedish beef herds was around 10 %. This is in agreement with a seroprevalence of 7.1 % determined in bulk-tank milk samples (BTM) in 205 Swedish dairy herds in 2008 [28], whereas it was 25 % in 426 herds examined in 2012 [15]. This indicates that the liver fluke burden was more or less the same in dairy and beef cattle in Sweden in 2007-2008. The apparent dramatic increase in seroprevalence in dairy herds which occurred between 2008 and 2012 suggests that a similar increase in *F. hepatica* abundance may occur in beef cattle. Although this needs further validation, it is supported by data from Swedish abattoirs showing an almost fourfold increase in *F. hepatica* prevalence in all slaughtered cattle (dairy and beef), from 3 % in 2005 to 11 % in 2013 [4].

The herd seroprevalence of *F. hepatica* infection in dairy cattle in European studies conducted between 2006 and 2008 was 37-40 % in Belgium [18], around 24 % in Germany [11] and 72-86 % in the UK [9]. Although the overall seroprevalence in Swedish beef cattle was lower than in these other Western Europe countries, the figures for the other European countries were obtained exclusively through BTM screening. Thus, there are differences in both sample collection method and animal category. It can also be argued that sampling only 1-5 animals per herd, as was done in the present study, is an insufficiently representative sample size. However, these samples represent the infection status at herd level [25]. Likewise, it has been demonstrated that at least 20-27 % of the herd must be infected to obtain a positive BTM response in ELISA [13, 29]. Although the use of individual blood samples rather than BTM could imply lower herd sensitivity, evidence of this is lacking. In addition, no other applicable methods for regional screening of beef cattle herds are available so far.

The scan cluster analysis identified two areas with a high risk of *F. hepatica* infection, both located in south-west Sweden. Similar clusters of infection have been reported in Belgium [8, 18]. Based on historical data from meat inspections, south-west Sweden was reported as having the highest abundance of bovine fasciolosis in the 1970s [30], which is in agreement with the present results. However, the reasons for this remain unclear.

According to the regression analyses, several factors contribute to the spatial distribution of *F. hepatica* in Sweden. In the final multivariate model, absence of coniferous forest and farm density were the major risk factors for *F. hepatica* exposure. Climate factors such as rainfall and temperature are essential for the transmission of *F. hepatica* and were previously considered to have a major impact on the risk of fasciolosis in ruminants [1]. The positive effects of rainfall and temperature on the survival and transmission of intermediate hosts and the larval stages of *F. hepatica* often explain the variation observed in the spatial distribution of *F. hepatica* in climate models [16, 31, 32]. However, although the climate is of fundamental importance for the spread of *F. hepatica*, recent reports show that climate factors surprisingly show a smaller association than those related to environment factors and herd management [11, 17, 18]. This was confirmed in the present study, where rainfall and temperature had only a minor effect in the multivariate analysis. Similarly, low or inconsistent associations between local/regional rainfall and *F. hepatica* distribution have been shown both in Australia [33] and in Germany [11]. In the present study, there was in fact a negative correlation between rainfall and *F. hepatica* seropositivity, in agreement with some previous studies [18, 34]. This indicates the complexity of the different contributing factors.

It has been suggested that certain soil types affect the existence, and thus the local distribution, of snail intermediate host habitats [25, 35]. Thus, the soil composition of minerals, its porosity, mineral content in water, pH and electrical conductivity have all been suggested to be of central importance [17, 21, 35, 36]. In the present study, Dystric Cambisol and Regosol soils were negative predictors, whereas Leptosol soil was a positive predictor. To our knowledge, there is only limited information available on the association between soil type (according to FAO classification) and distribution of freshwater molluscs [37]. A total of seven different soil types are present in Sweden and some of these overlap in many areas [38]. The association between soil type and *F. hepatica* seropositivity in our model seems to be a reflection that specific soils (Cambisols, Regosols, Leptosols) are present or absent in the two clusters. For instance, Leptosols are frequently found in south-west Sweden, where most *F. hepatica* seropositive farms also were found. Thus, the suggestion of a relationship between intermediate hosts of *F. hepatica* or environmental stages of liver fluke and soil type remains rather speculative at this stage. However, there were strong associations between certain soil mineral concentrations and herd *F. hepatica* seropositivity, with Cu and P being negatively associated and Fe positively associated. Copper is known for its toxic effect on lymnaeid snails [39], whereas the results for Fe and P contradict those in a UK study [10]. It has been suggested that certain soils and minerals are more suitable for snails than others [21, 40]. As with soil types, it appears that basing prediction of fasciolosis outbreaks on soil minerals remains debatable. Although this requires further investigation, it may be speculated that the living conditions for the main intermediate host are influenced both by soil type and minerals.

South-west Sweden is the most intensive agricultural region in the Scandinavian Peninsula, with a high proportion of grazing livestock. After the Scandinavian mountain range along the Norwegian-Swedish border, the west coast has the next highest rainfall in Sweden, up to 1000-1200 mm per year (SMHI: http://www.smhi.se/kunskapsbanken/klimat/sveriges-klimat-1.6867). Together with the relatively warm Atlantic climate, this provides suitable conditions for the survival and transmission of *F. hepatica* and its intermediate host in south-west Sweden. Cluster 1 (in Västra Götaland) was located on the Atlantic coast, in the area with optimal conditions, whereas Cluster 2 (in Skåne) lay outside the rainiest region (Additional file 6: Figure S1). However, the area between Clusters 1 and 2 on the west coast (in Halland) had a relatively low seroprevalence, despite being densely populated by cattle. This suggests that the situation is complex and that several other factors apart from rainfall determine the spatial distribution of *F. hepatica*.

Most cattle in Sweden are raised in the central and south-western areas of the country and there was some association between the number of animals and *F. hepatica* seroprevalence. However, one positive herd was also found in Byske near latitude 65° (GPS 64.953845, 21.204164). To our knowledge, this is the most northerly occurrence of *F. hepatica* reported to date in the world. Although the possibility that the *F. hepatica*-seropositive animal in question was imported from other parts of Sweden cannot be excluded, repeated findings of liver fluke in the period 2008-2012 at local abattoirs in Norrland [4] confirm the presence of *F. hepatica* in this subarctic area. The fact that *F. hepatica* can complete its life cycle at this latitude, only approximately 200 km south of the Arctic Circle, is supported by data from the nearest meteorological station. It has been suggested that *F. hepatica* can complete its life cycle when the temperature exceeds 10 °C for a minimum of 2 months per year, enabling intramolluscan development [1]. In Byske, the mean temperature exceeds 10 °C for approximately 3 months of the year (June = 11.1 °C; July = 14.5 °C; August = 13.4 °C) (http://www.smhi.se/klimatdata/meteorologi/2.1240).

The pseudo-R^2^ values for models with specific subsets varied from 5.9 to 9.6 % (Additional file 2: Table S2, Additional file 3: Table S3, Additional file 4: Table S4, Additional file 5; Table S5), while the final multivariate model explained 15.8 % of the variation in *F. hepatica* distribution. This agrees with a German study [11], but is considerably lower than the variation explained by models for England and Wales [10]. The low pseudo-R^2^ values in the present study indicate that important factors affecting the distribution of *F. hepatica* in Sweden have not yet been identified. These may include pasture management, which seemed to be a stronger predictor of *F. hepatica* infection than climate and environmental factors in Belgium [17, 18].

## Conclusions

The seroprevalence of *F. hepatica* infection in beef cattle herds in Sweden is described for the first time in this study. The spatial distribution map revealed two high-risk areas, both located in south-west Sweden. Several of the variables tested were associated with seropositivity in the sampled animals, e.g. the spatial distribution was influenced by multi-factorial effects with strong associations to cattle density, absence of coniferous forest and soil chemistry. However, no single predictor for forecasting of *F. hepatica* infection in Sweden was identified.

## Additional files

Additional file 1: Table S1. Bivariate logistic regression between variables and *Fasciola hepatica* seropositivity. Abbreviations: ^a^ number of animals/farms per km^2^. ^b^ 433 out of 2030 postcode areas (PSA) analysed were missing. * Significant at 0.05 level; ** Significant at 0.01 level; *** Significant at 0.001 level.

Additional flie 2: Table S2. *Model Temperature*: Results of multivariate analysis of the temperature dataset by logistic regression. * Significant at 0.05 level; ** Significant at 0.01 level; *** Significant at 0.001 level.

Additional file 3: Table S3. *Model Precipitation*: Results of multivariate analysis of the precipitation dataset by logistic regression. * Significant at 0.05 level; ** Significant at 0.01 level; *** Significant at 0.001 level.

Additional file 4: Table S4. *Model Corine Land Cover*: Results of multivariate analysis of the land cover dataset by logistic regression. * Significant at 0.05 level; ** Significant at 0.01 level; *** Significant at 0.001 level.

Additional file 5: Table S5. *Model Minerals in Soil*: Results of multivariate analysis of the land cover dataset by logistic regression. * Significant at 0.05 level; ** Significant at 0.01 level; *** Significant at 0.001 level.

Additional file 6: Figure S1. Mean rainfall (10-year average) during the growing season (April-October) and distribution of *Fasciola hepatica-*positive beef cattle herds in south-west Sweden.
