# Supplementary figures and images for: Impact of meteorological and environmental factors on the spatial distribution of Fasciola hepatica in beef cattle herds in Sweden
Source: BMC Vet Res. 2015 Jun 9;11:128. doi: 10.1186/s12917-015-0447-0 (PMC4459679; doi:10.1186/s12917-015-0447-0)

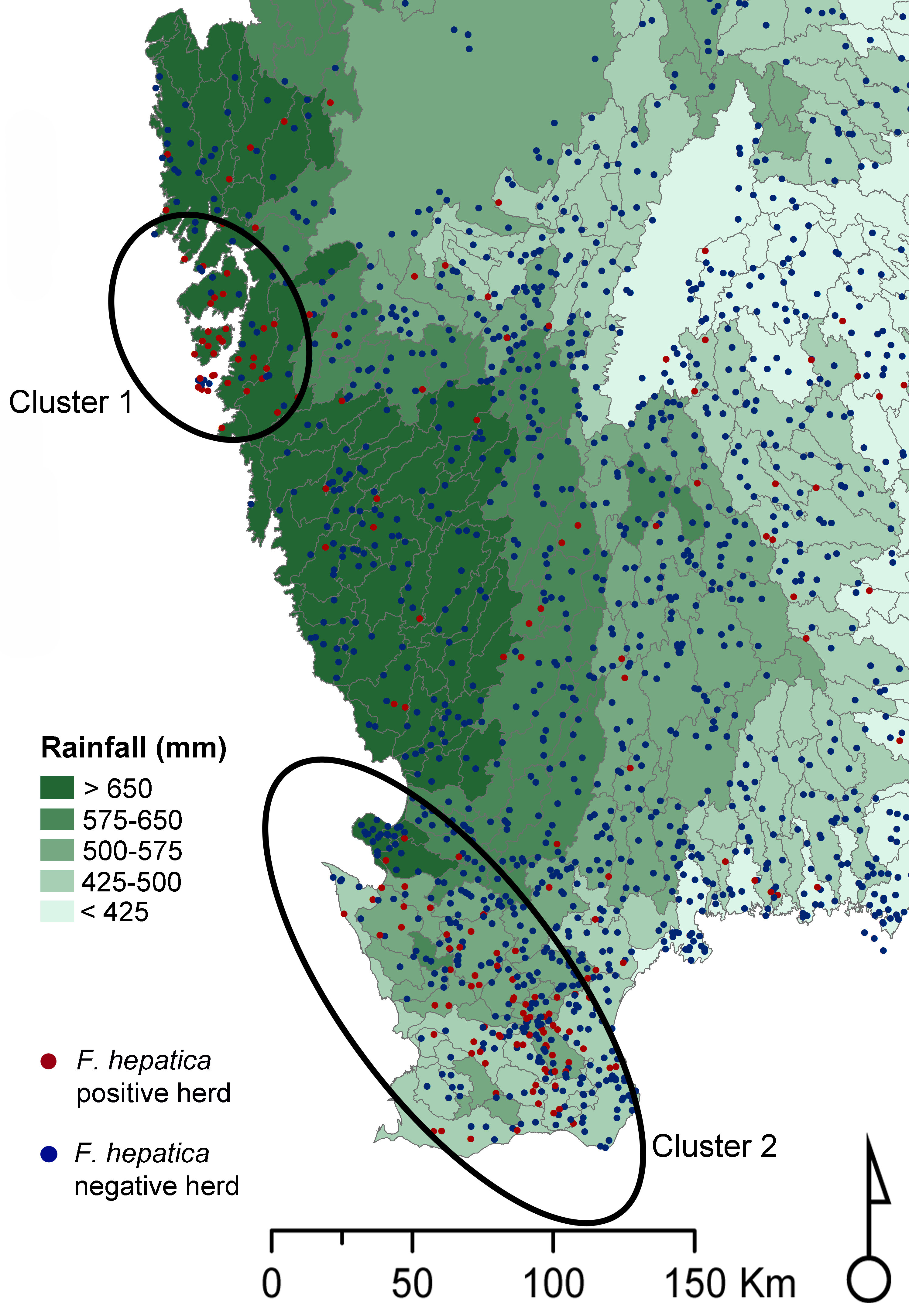

Supplement: Supplementary file 6 — Mean rainfall (10-year average) during the growing season (April-October) and distribution of Fasciola hepatica-positive beef cattle herds in south-west Sweden. [file 12917_2015_447_MOESM6_ESM.png]
